# Gut microbiota dysbiosis induced by alcohol exposure in pubertal and adult mice

**DOI:** 10.1128/msystems.01366-24

**Published:** 2024-11-27

**Authors:** Jinlong Yang, Haoyu Wang, Xiaoqian Lin, Jincen Liu, Yue Feng, Yuyin Bai, Hewei Liang, Tongyuan Hu, Zhinan Wu, Jianghua Lai, Jianmei Liu, Yuanqiang Zou, Shuguang Wei, Peng Yan

**Affiliations:** 1 College of Forensic Science, Xi’an Jiaotong University, Xi’an, Shaanxi, China; 2 BGI Research, Kunming, China; 3 BGI Research, Shenzhen, China; 4 College of Life Sciences, University of Chinese Academy of Sciences, Beijing, China; 5 School of Basic Medicine, Qingdao Medical College, Qingdao University, Qingdao, China; 6 School of Biology and Biological Engineering, South China University of Technology, Guangzhou, China; 7 NHC Key Laboratory of Forensic Science, Xi’an Jiaotong University, Xi’an, Shaanxi, China; 8 BGI Research, Wuhan, China; 9 Shenzhen Engineering Laboratory of Detection and Intervention of Human Intestinal Microbiome, BGI Research, Shenzhen, China; University of California, San Francisco, San Francisco, California, USA

**Keywords:** gut microbiota, alcohol, metagenomics, puberty, adulthood, intestinal barrier

## Abstract

Alcohol intake causes many diseases including neuropsychiatric symptoms, nutritional deficiency, progressive pancreatitis, liver cirrhosis, and ischemic heart disease. The gut microbiota changes significantly after alcohol exposure. Alcohol consumption tends to increase in underage and young people, but the feature of the gut microbiota in puberty remains largely unexplored. In this study, we conducted alcohol-exposed pubertal and adult mice model to investigate the intestinal damage and gut microbiota change. Interestingly, the responses of pubertal mice and adult mice after alcohol exposure were different. We found that alcohol dehydrogenase decreased and aldehyde dehydrogenase increased in the liver of pubertal mice, thus reducing the accumulation of toxic acetaldehyde. Furthermore, alcohol exposure caused less intestinal injury in pubertal mice. Through the analysis of metagenome assembly genome, we obtained many unrecognized bacterial genomes. *Limosillactobacillus reuteri* (cluster_56) and *Lactobacillus intestinalis* (cluster_57) were assembled from the samples of pubertal mice, which were involved in the production of indole acetic acid and the transformation of bile acids in response to alcohol exposure. This study provided a new insight to investigate the gut microbiota change and explained the difference of the gut microbiota after alcohol exposure between pubertal mice and adult mice.

**IMPORTANCE:**

This study elucidates the significant impact of alcohol exposure on the gut microbiota and metabolic pathways in mice, highlighting the differential responses between adolescent and adult stages. Alcohol exposure was found to damage the intestinal barrier, alter the microbial composition by decreasing beneficial bacteria like *Lactobacillus*, and increase harmful bacteria such as *Alistipes*. The study also discovered unique microbial changes and resilience in pubertal mice. Species-level metagenomic analysis revealed specific microbial taxa and metabolic functions affected by alcohol. Metagenome-assembled genomes (MAGs) found many species that could not be annotated by conventional methods including many members of *Lachnospiraceae*, greatly expanding our understanding of the gut microbiota composition. These findings underscore the need for further research on alcohol’s effects on various organs and the implications of microbial metabolites on disease progression.

## INTRODUCTION

Alcohol is one of the most prevalent sources of non-infectious diseases, culminating in approximately 3.3million fatalities annually, which constitutes 6% of all mortality worldwide (1, 2). Chronic alcohol abuse constitutes a primary etiological factor for early death, causing excessive harmful effects on various physiological systems (3–6). These encompass neuropsychiatric manifestations, nutritional deficiencies, progressive pancreatic inflammation, cirrhosis, and ischemic cardiac conditions (7–9). A Mendelian randomized study confirmed that daily drinking, even mild drinking, significantly increased the risk of hypertension and coronary artery disease (10). With the popularity of alcoholic beverages, the amount and frequency of alcohol drinking among teenagers have also increased significantly (11).

The close correlation between gut microbiota and various diseases has been widely reported, and both acute and chronic alcohol intake can change the structure and function of gut microbiota, suggesting the gut microbiota may serve as a bridge between alcohol intake and the occurrence of various diseases (12, 13). A cohort study showed that the abundance of *Faecalibacterium praustnizii* significantly decreased and *Lachnospiraceae* increased after alcohol exposure (14). Similarly, alcohol intake also significantly reduced the relative abundance of *Faecalibacterium* and *Akkermansia* and increased bacterial translocation in mice model (15). These studies based on 16S rRNA sequencing partly revealed the changes of gut microbiota after alcohol exposure but still had some limitations. Previous studies have also shown that supplementing probiotics can regulate the gut microbiota and improve intestinal barrier damage caused by alcohol intake, suggesting that gut microbiota may play a crucial role in this process (16). Therefore, studying the structural and functional changes of gut microbiota after alcohol exposure has become more important.

There are differences in physiological changes after alcohol intake between adolescents and adults (17–20). Specifically, adolescents are less sensitive to the toxic effects of alcohol, such as uncoordinated movement and social disorders (11). Interestingly, a study of 234 adolescents found that drinking was positively correlated with memory, even when age, socioeconomic status, abstinence, gender, and baseline performance were controlled (21). On the contrary, alcohol intake in adults, whether chronic or acute, will lead to serious damage to the body and negative effects on the nervous system (22). In view of the large differences in physiological changes between the two after alcohol exposure, there is a lack of high-resolution metagenomic research on the differences of the gut microbiota between adults and adolescents.

In this study, we conducted alcohol-exposed mice model to investigate the change of gut microbiota. The results showed that pubertal mice were less sensitive to alcohol-induced intestinal injury and mucin secretion reduction compared with adult mice. By employing metagenomic assembly and binning methods, we discovered many new species in the alcohol-treated groups, and the vast majority of taxonomically unknown taxa also responded strongly to alcohol exposure, which may have been overlooked by previous reference-based analysis methods. Our results provide important evidence for further study on the relationship between alcohol exposure and gut microbiota.

## MATERIALS AND METHODS

### Animals

Male C57BL/6J mice (3 and 7 weeks old) were purchased from Beijing Vital River Laboratory Animal Technology (Beijing, China) and were housed in pathogen-free rooms in groups of four under controlled conditions (12-hour light/dark cycle, lights on at 8:00 a.m., 50% ± 5% humidity, and 22 ± 3°C temperature control). The animals were provided with free access to sterile water and were fed *ad libitum* with AIN-93 standard diet. All mice were acclimated to the environment for 7 days and were handled daily for 4 days before the experiment.

### Drug administration and fecal sample collection

**Fig 1:**
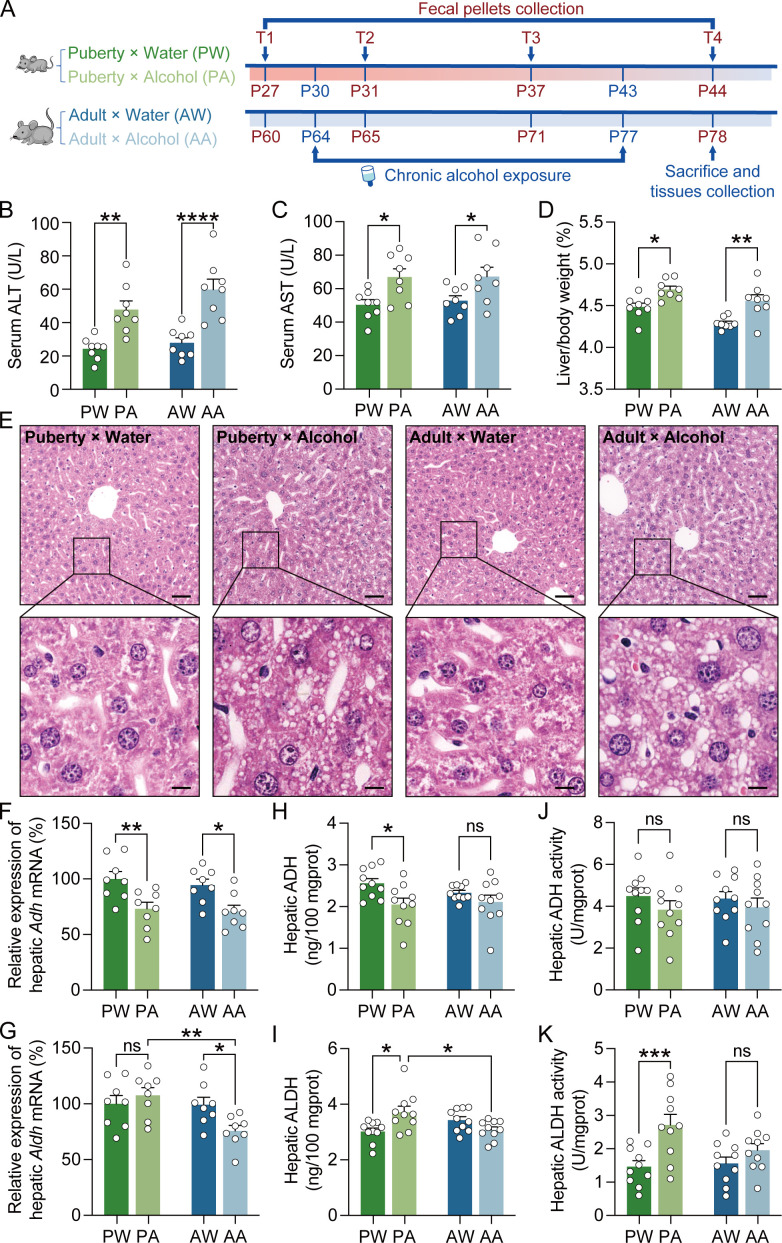
Liver injury and hepatic ADH and ALDH changes after chronic alcohol exposure. (**A**) An overview of the experimental timing. Fecal samples were collected at four time points: before alcohol exposure (**T1**), 24 hours after alcohol exposure (**T2**), 1 week after alcohol exposure (**T3**), and 2 weeks after alcohol exposure (**T4**). ALT (**B**) and AST (**C**) levels in serum (*n* = 8). (**D**) The ratio of liver/body weight (*n* = 8). (**E**) Representative images of HE staining for liver sections. The boxes indicate regions shown at higher magnification in the lower panels; scale bars represent 50 µm under low magnification and 10 µm under high magnification (*n* = 5). The relative mRNA expression (**F, G**), content (**H, I**), and activity (**J, K**) of ADH and ALDH in the liver (*n* = 8, 10). Data are presented as the mean ± SEM, each symbol represents the independent of a single animal; two-way ANOVA followed by the Bonferroni post hoc test. Significance levels: *, *P <* 0.05; **, *P <* 0.01; ***, *P <* 0.001; ****, *P <* 0.0001; ns, not significant.

Food-grade alcohol (75% vol/vol) was dissolved in sterile water to a final concentration of 20% vol/vol. Alcohol solution was freshly prepared every single day. All pubertal and adult mice were randomly divided into water or chronic-alcohol-exposed group, and were given sterile water or alcohol solution (20% vol/vol) as their sole liquid source during the dark cycle for 2 weeks (puberty: PND 30~43, adult: PND 64~77). Fecal samples were collected at four time points: before alcohol exposure (T1), 24 hours after alcohol exposure (T2), 1 week after alcohol exposure (T3), and 2 weeks after alcohol exposure (T4), with each fecal collection at 8:00 p.m. An overview of the experimental timing is provided in Fig. 1A.

### Determination of serum ALT and AST and hepatic ADH and ALDH

At the end of the experiment, the blood and liver samples were collected. The alanine aminotransferase (ALT) and aspartate aminotransferase (AST) levels in mice serum were detected by the Mouse Aspartate Aminotransferase ELISA Kit (Cusabio Biotech, Cat# CSB-E12649m) and Mouse Alanine Aminotransferase ELISA Kit (Cusabio Biotech, Cat# CSB-E16539m) following the manufacturer’s instructions.

The mRNA expression of hepatic *Adh* and *Aldh* was analyzed by quantitative PCR (qPCR). The total RNA was extracted from the liver. cDNA was synthesized by PrimeScript RT Master Mix (Takara, Cat# RR036). qPCR was performed on a CFX96 Touch Real-Time PCR Detection System (Bio-Rad) using TB Green Premix Ex Taq (Takara, Cat# RR420). Relative expression levels were normalized against *Gapdh* and were calculated using the 2^−ΔΔ^Ct method. Primer sequences are listed in Table S1.

The content of alcohol dehydrogenase (ADH) and aldehyde dehydrogenase (ALDH) in mice liver was quantified by the Mouse Alcohol dehydrogenase ELISA kit (Jonln, Cat# JL14053) and Mouse Acetaldehyde dehydrogenase ELISA kit (Jonln, Cat# JL20658) following the manufacturer’s instructions.

The activity of ADH and ALDH in mice liver was analyzed by the Alcohol Dehydrogenase Activity Assay Kit (Solarbio Life Sciences, Cat# BC1085) and Acetaldehyde Dehydrogenase Activity Assay Kit (Solarbio Life Sciences, Cat# BC0755) according to the manufacturer’s instructions.

### Histological analysis

On the endpoint of the experiment, the livers and small intestines (prepared as Swiss rolls) were fixed overnight in 4% paraformaldehyde. After dehydration with an alcohol gradient, the tissues were embedded in paraffin and sectioned into 4 µm. Subsequently, hematoxylin and eosin (H&E) and Alcian blue-periodic acid Schiff (AB-PAS) staining were performed according to the manufacturer’s protocol (H&E: Servicebio, Cat# G1005; AB-PAS: Servicebio, Cat# G1049).

For the small intestine, each Swiss roll was divided into three sections: proximal (duodenum), medial (jejunum), and distal (ileum), for histological analysis in a blinded manner, independently by two researchers. Twenty villi, crypts, and goblet cells on the villi were randomly analyzed in each section, and the length of villi, depth of crypts, and number of goblet cells were recorded. In addition, the degree of mucosal damage was evaluated using a histological scoring system as described previously with some modifications (Table S2) (23).

### DNA extraction, sequencing, and preprocessing

Total genomic DNA for library preparation and metagenomic sequencing was extracted using an MGIEasy Microbiome DNA Extraction Kit (MGI Tech Co., Ltd.). Paired-end 100 bp sequencing using a DNBSEQ-T7 platform produced an average of 130,834,806 raw sequence reads per sample. Adapter sequences were removed, and the raw sequence reads were quality filtered using fastp (24) v0.23.2 with options “--trim_poly_g --poly_g_min_len 10 --trim_poly_x --poly_x_min_len 10 --cut_front --cut_tail --cut_window_size 4 --cut_mean_quality 20 --qualified_quality_phred 15.” Host sequences were removed after mapping the Mus musculus genome GRCm39 (GCF_000001635.27) using Bowtie 2v2.4.5 (25) to obtain an average of 118,772,466 clean reads per sample.

### Taxonomic analysis of the metagenomes

The clean reads were mapped to the Kraken database (k2_pluspfp_20220908) by Kraken2 v2.1.2 (26), a k-mer-based tool, to generate a taxonomic profile. The results of Kraken2 were processed with Bracken v2.5 (27). Relative abundance in a sample is defined as the number of assigned reads compared with the number of total clean reads.

The R package “vegan” was used to evaluate the alpha and beta diversity, specifically, the Shannon index and Bray-Curtis dissimilarity based on the relative abundance profiles were calculated by the functions “diversity” and “vegdist.”

Linear discriminant analysis (LDA) effect size (LEfSe) (28) analysis was performed using the Huttenhower Lab Galaxy Server, to identify the feature that had significant differences between the two groups. Results were shown with an LDA score above 2.00 and *P* < 0.05 for the factorial Kruskal–Wallis test. The co-occurrence analysis of significant enriched bacteria was conducted by R function “corr.test” and visualized with Cytoscape10 v3.9.1.

### Functional analysis of the metagenomes

For each group of samples at the experimental endpoint, general functional annotation was performed with the HUMAnN3 v3.5 (29). The abundance of the MetaCyc pathway was determined using the HUMAnN3 tool based on the official database released in 201901. The R package “DESeq2” and “ALDEx2” were used to calculate the difference in the abundance of pathways between the two groups.

Next, the clean reads obtained were assembled into contigs using MegaHit v1.2.9 (30). Gene ORFs for the assemblies were predicted using Prodigal v2.6.3 (31) with the parameters -m -p meta. The complete ORFs were extracted and de-replicated by CD-HIT v4.8.1 (32) utilizing specific parameters such as -c 0.95 and -aS 0.8, which defined stringent criteria of 95% identity and 80% coverage. An index was built for the resulting gene catalog using Bowtie 2v2.4.5 (25). Quality control metagenomic reads per sample were quantified using featureCounts v2.0.3 (33) to calculate transcript per million (TPM). In addition, the functions of the de-replicated gene catalog were annotated against the Kyoto Encyclopedia of Genes and Genomes (KEGG) database using eggNOG-mapper v2 (34) with eggNOG database version 5.0.2.

### Metagenome binning, quality control, and clustering

Assembled contigs of each sample were binned using MaxBin2 v2.2.6 (35), MetaBAT2 v2.12.1 (36), and CONCOCT v1.0.0, respectively, with default parameters. Completeness and contamination of metagenome-assembled genomes (MAGs) were calculated using the “lineage_wf” module of CheckM v1.1.2 (37). As a result, MAGs that meet the quality requirements (completeness >90% and contamination <10%) were selected as the high-quality MAGs. Finally, the high-quality MAGs were clustered into 315 representative species-level genome bins (SGBs) using dRep v3.4.0 (38) based on a genome-wide average nucleotide identity (ANI) threshold of 95%.

### Comparison SGBs to public mouse microbiota-derived bacterial genomes

The representative genomes for the 1,094 species of the Mouse Gastrointestinal Bacterial Catalogue (MGBC) were downloaded from the European Nucleotide Archive (ENA) under project accession PRJEB45234. FastANI v1.33 (39) was used to compare the 315 SGBs against each MGBC representative genomes. The SGBs were considered to match if the pairwise ANI value was higher than 95%.

### Taxonomy annotation, abundance analysis, and function annotation

Genome Taxonomy Database Toolkit (GTDB-Tk, version 2.1.0) (40) with reference to GTDB release R20724 was used to perform taxonomic annotation of each SGBs and reconstruct the maximum-likelihood phylogenetic tree based on 120 conserved single-copy genes. The phylogenetic tree was viewed using the display and annotation tool iTOL v6 (41).

To enable species-resolved metagenomic analysis, we built a custom Kraken2/Bracken database with 315 SGBs. Kraken2 v2.1.2 and Bracken v2.5 were also used to calculate the read numbers of the 315 SGBs in each sample. We defined more than 0.01% of reads for the assigned classification as the presence of SGB in the sample.

The coding sequence (CDS) of the 315 SGBs was predicted using Prokka v1.14.6 (42), and KEGG was annotated using eggnog-mapper v2 with eggNOG database version 5.0.2 (34).

### Statistical analysis

The enzyme-linked immunosorbent assay (ELISA), qPCR, and histological data were analyzed by two-way analysis of variance (ANOVA) using GraphPad Prism 9 Software (GraphPad Software, San Diego, CA, USA). Bonferroni post hoc analysis was used when appropriate. One-way ANOVA was used for data that met the requirement of being normally distributed, and Kruskal–Wallis test was used for data that did not meet this requirement. Wilcoxon test was used to compare two groups. Statistical significance was set at *P* < 0.05. Detailed statistics are provided in Table S3.

## RESULTS

### Liver injury and hepatic ADH and ALDH changes after chronic alcohol exposure

Serum ALT and AST levels are associated with the degree of liver injury. Our results demonstrate that both pubertal and adult alcohol-exposed groups had significantly increased serum ALT and AST levels compared with the water group (Fig. 1B and C), as well as increased liver/body weight ratio (Fig. 1D). Histological analysis via H&E staining confirmed that alcohol exposure led to obvious liver pathology, primarily featuring a rise in small lipid droplets (Fig. 1E). ADH and ALDH in the liver are directly related to alcohol metabolism. Alcohol exposure significantly reduced hepatic *Adh* mRNA expression in both pubertal and adult mice, while hepatic *Aldh* mRNA expression was reduced only in the adult mice (Fig. 1F and G). ELISA analysis showed that hepatic ADH protein content was significantly reduced and hepatic ALDH protein content increased in the pubertal alcohol-exposed group, with no significant changes observed in the adult alcohol-exposed group (Fig. 1H and I). Additionally, no significant differences in hepatic ADH activity were found between groups, while hepatic ALDH activity was significantly higher in the pubertal alcohol-exposed group compared with the water group (Fig. 1J and K). These findings suggest that alcohol exposure at different life stages results in distinct changes in liver enzyme expression and activity, influencing the liver’s ability to metabolize alcohol.

### Different intestinal injuries in pubertal and adult mice

**Fig 2:**
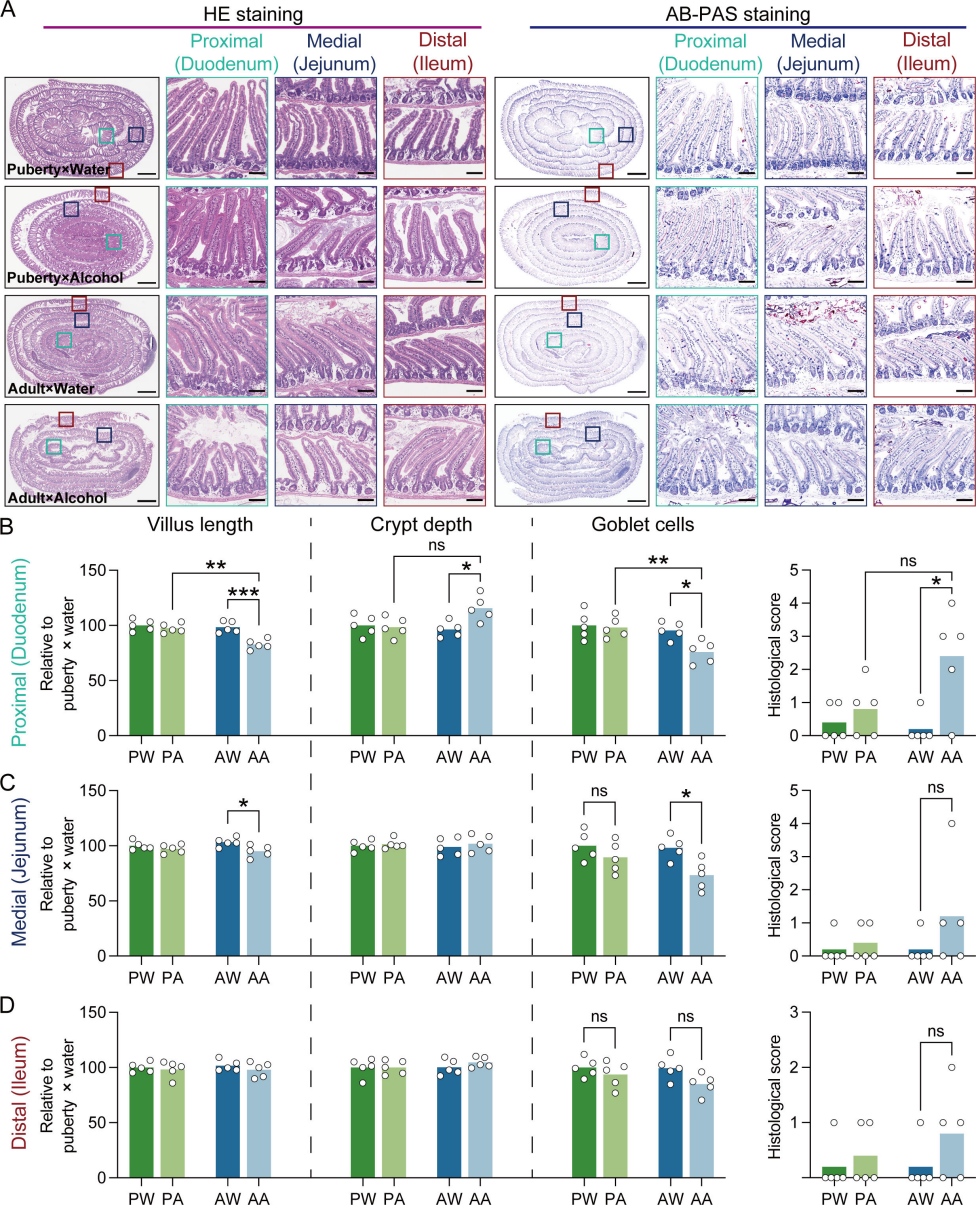
Intestinal injury and histological score. (**A**) Representative images of histology images stained with H&E (left) and AB-PAS (right). The small intestines were divided into three sections for analysis: proximal (duodenum), medial (jejunum), and distal (ileum). The boxes indicate regions shown at higher magnification in the lower panels; scale bars represent 1,000 µm under low magnification and 100 µm under high magnification. The relative changes in the length of villi, depth of crypts, and number of goblet cells, and the histologic scoring were analyzed in the duodenum (**B**), jejunum (**C**), and ileum (**D**), respectively (*n* = 5). Data are presented as the mean ± SEM, each symbol represents a single animal; two-way ANOVA followed by the Bonferroni post hoc test. Group: PW, pubertal mice in the water group; PA, pubertal mice in the alcohol-exposed group; AW, adult mice in the water group; AA, adult mice in the alcohol-exposed group. Significance levels: *, *P* < 0.05; **, *P* < 0.01; ***, *P* < 0.001; ns, not significant.

After alcohol exposure, H&E staining and AB-PAS staining were examined in three different positions (duodenum, jejunum, and ileum) (Fig. 2A). In duodenum, we found that four indicators, including villus length, goblet cells, crypt depth, and histological score, which reflect intestinal function, did not deteriorate in the pubertal mice group (Fig. 2B; Table S2). Conversely, after consuming the same amount of alcohol, these indicators deteriorated in the adult mice group and became worse. The same situation happened in jejunum (Fig. 2C; Table S2). In ileum, the alcohol-induced intestinal dysfunction almost disappeared (Fig. 2D; Table S2). These results indicated that the closer to the upper gastrointestinal tract, the greater the injury caused by alcohol. Meanwhile, the tolerance of the pubertal mice to alcohol was better than that of the adult mice.

### Alcohol-induced alterations in gut microbiota composition in adult mice

**Fig 3:**
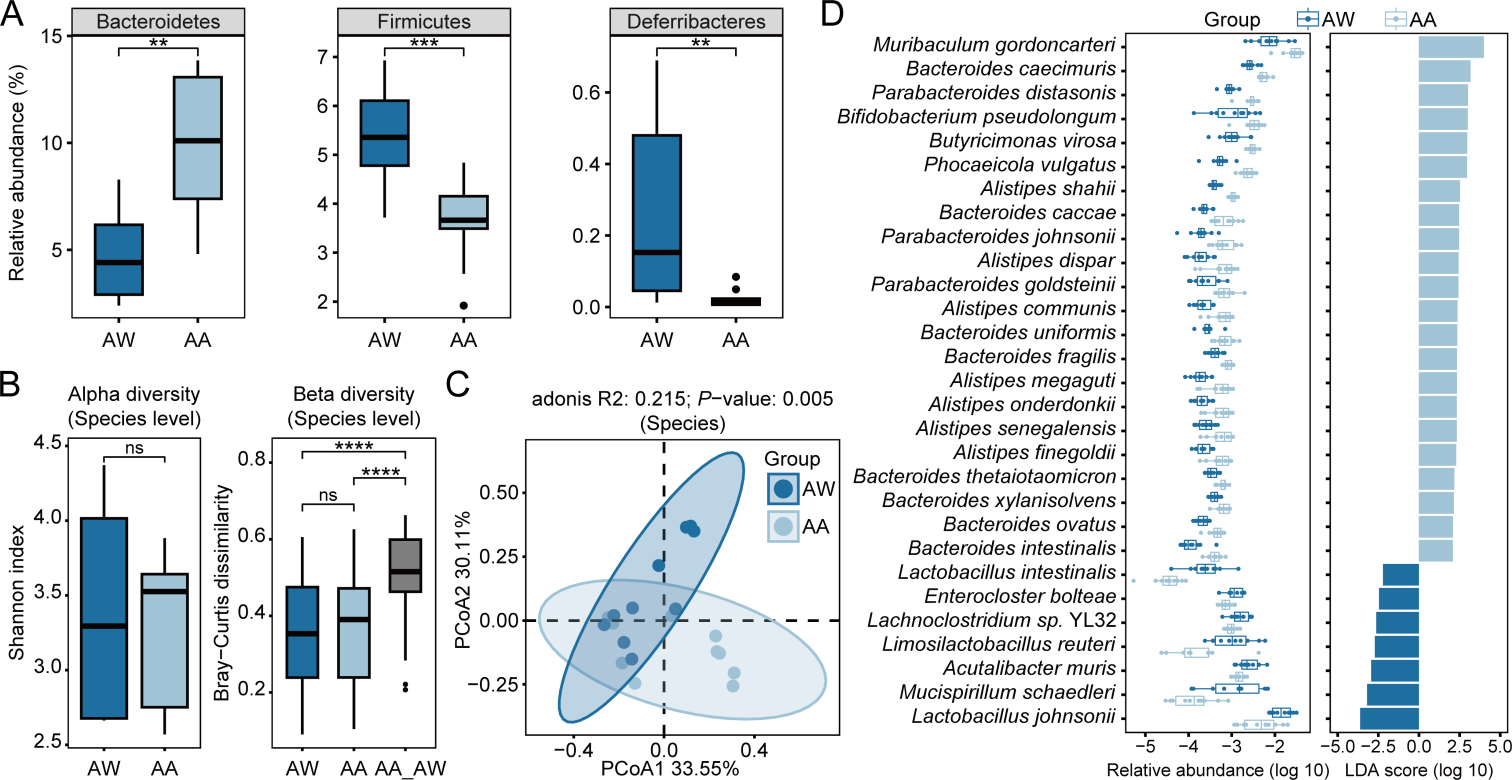
Alterations of the gut microbiota in adult mice caused by alcohol exposure. (**A**) Relative abundances of Bacteroidetes, Firmicutes, and Deferribacteres. (**B**) Shannon index and Bray-Curtis dissimilarity of gut microbiota at the species level from each group of mice. (**C**) PCoA of the gut microbiota from each group of mice. (**D**) Twenty-nine species with significantly different abundances in the water group and alcohol-exposed group. The left panel shows the relative abundance of the species in each group, and the right panel shows the LDA score. Group: AW, adult mice in the water group; AA, adult mice in the alcohol-exposed group. Significance levels: *, *P <* 0.05; **, *P <* 0.01; ***, *P <* 0.001; ns, not significant.

To investigate the impact of alcohol exposure on the gut microbiota, we conducted a shotgun metagenomic sequencing on a total of 80 samples obtained from 20 adult mice across four exposure periods. This analysis showed that the predominant bacterial phyla constituting the majority of the microbiome in each group of mice were Verrucomicrobia, Bacteroidetes, and Firmicutes, accounting for an average of 41.40%, 36.66%, and 16.44% of the bacterial sequences, respectively (Fig. S1A). Compared with the water group, the alcohol-exposed group led to a significant decrease in the relative abundance of Firmicutes and Deferribacteres while increasing the relative abundance of Bacteroidetes (Fig. 3A).

At the genus and species levels, we observed that alcohol exposure did not significantly change the diversity of gut bacteria (Fig. 3B; Fig. S1B), but it did impact the community composition. At the experimental endpoint, the principal coordinate analysis (PCoA), utilizing Bray-Curtis distances of metagenomic sequencing profiles at the genus and species levels, unveiled significant distinctions between the alcohol-exposed group and the water group (Fig. 3C; Fig. S1C). A total of eight genera were detected to be enriched in the alcohol-exposed group, while another six genera were enriched in the water group. Notably, in the alcohol-exposed group, the relative abundance of potentially beneficial bacteria genera, such as *Lactobacillus* and recently reclassified *Limosilactobacillus*, decreased, while the relative abundance of the harmful genus *Alistipes* (43) increased (Fig. S1D). Furthermore, our analysis revealed an enrichment of species associated with infections in the alcohol-exposed group, including *Butyricimonas virosa* (44), *Phocaeicola vulgatus*, *Alistipes shahii*, *Bacteroides caccae*, *Parabacteroides goldsteinii*, *Bacteroides fragilis*, *Alistipes onderdonkii*, *Alistipes finegoldii*, and *Bacteroides ovatus* (Fig. 3D). Conversely, the abundance of *Lactobacillus* spp. and gut-protective species exhibited a significant decrease (Fig. 3D). The mucus layer, which constitutes the second chemical barrier within the intestine, serves as a defense mechanism against enteric pathogens. Notably, *Mucispirillum schaedleri*, the sole representative of Deferribacteres colonizing the mucus layer of the rodent gastrointestinal tract (45), has the ability to assist the host in resisting intestinal inflammation caused by *Salmonella*, achieved through the inhibition of *Salmonella* virulence factors expression. Therefore, the reduction of *Mucispirillum schaedleri* concurrent with alcohol exposure carries the risk of inflammation. Importantly, alcohol exposure led to a stronger association among these infection-associated species, with *Alistipes* and *Bacteroides* in particular showing a large number of significant positive associations (Fig. S1E).

We observed four different exposure periods and found that the alpha diversity of intestinal microorganisms in adult mice exhibited a trend of initial decrease, followed by an increase during the observation period (Fig. S2A). Acute exposure for 24 hours had a significant impact on both community diversity and composition (Fig. S2B and C). After 24 hours of acute exposure, the intestinal microbial composition of the water group tended to stabilize, while the composition of the alcohol-exposed group underwent substantial changes, deviating from the baseline period (Fig. S2D). These findings indicated a pronounced response of gut microbes to 24-hour alcohol exposure, shaping distinct microbial communities thereafter.

### Impaired gut microbiota function as a result of alcohol consumption in adult mice

In order to explore the functional changes in the gut microbiota during alcohol exposure, we employed HUMAnN3 to assess the functional capacity of the gut metagenome. Using the default MetaCyc pathway database, we calculated the relative abundance of each metabolic pathway in the samples. Our analysis of metabolic pathways revealed that alcohol exposure resulted in significant reductions in 23 processes, such as superpathway of glucose and xylose degradation, lactose and galactose degradation I, pyruvate fermentation to acetate and (S)-lactate I, pyruvate fermentation to acetate and lactate II, GABA shunt, and 4-aminobutanoate degradation V (Fig. S3A). Conversely, we observed an enrichment of pathways associated with the urea cycle, superpathway of GDP-mannose-derived O-antigen building blocks biosynthesis, biotin biosynthesis, and fatty acid elongation-saturation (Fig. S3A). These changes suggest alterations in energy metabolism and nutrient utilization within the gut microbiota.

Alcohol can damage the intestinal mucosal barrier through its own damaging effect on intestinal epithelial cells and its metabolites distributed in the blood, and the intestinal microbiota is involved in regulating the integrity and function of the intestinal barrier. To investigate changes in the abundance of genes associated with gut protection, we processed and compiled clean reads from all endpoint samples, enabling the prediction of protein-coding genes. This process facilitated the construction of a 1.33-M non-redundant catalog of gut microbial genes. Subsequently, the reads were mapped to the gene catalog for each individual sample, generating a gene profile, and gene annotation was performed using the KEGG.

**Fig 4:**
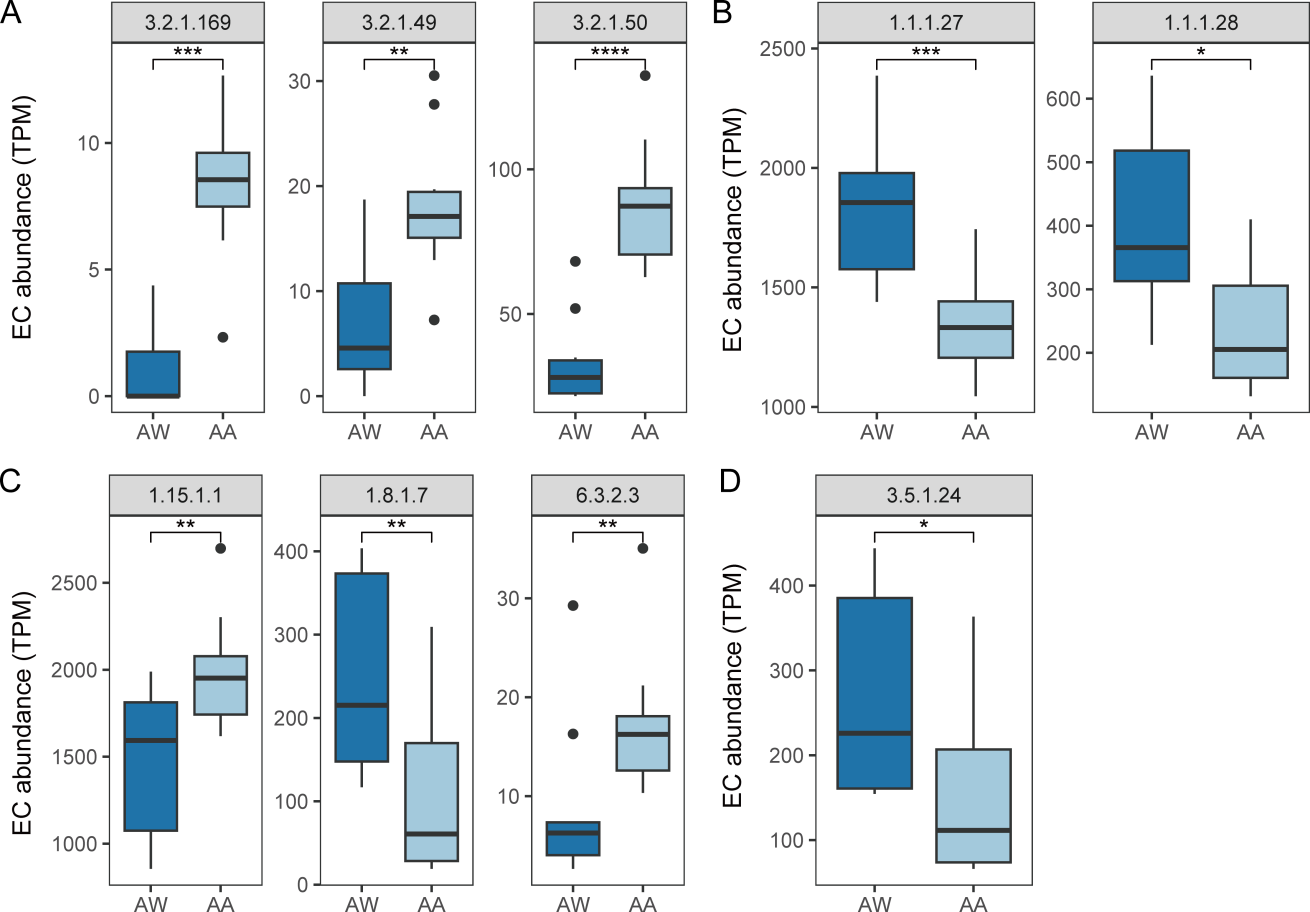
Comparison of the abundance of enzymes involved in (**A**) mucin degradation, (**B**) oxidative stress, (**C**) tryptophan to indoleacetic acid, and (**D**) bile acid conversion in the two groups. TPM (transcript per million) is defined as abundance. Group: AW, adult mice in the water group; AA, adult mice in the alcohol-exposed group. Significance levels: *, *P <* 0.05; **, *P <* 0.01; ***, *P <* 0.001; ns, not significant. EC: Enzyme Nomenclature (EC number system).

Intriguingly, the analysis of gene abundance associated with gut protection highlighted significant increases in enzymes related to mucin degradation in the alcohol-exposed group (Fig. 4A). This finding implies a potential disruption of the intestinal mucus layer barrier, which could have implications for gut health and inflammation. On the other hand, we observed a decrease in the conversion of bile acids and a reduction in the synthetase responsible for indole acetic acid production in the alcohol-exposed group (Fig. 4C and D), indicating possible disruptions in gut homeostasis and anti-inflammatory mechanisms.

Moreover, the alcohol-exposed group exhibited an increase in the abundance of superoxide dismutase and glutathione synthase (Fig. 4B), suggesting an adaptive response to enhance antioxidant protection, likely due to oxidative stress induced by alcohol exposure. However, there were no significant differences observed in butyrate production.

### Pubertal gut microbiota exhibits limited changes in response to alcohol consumption

**Fig 5:**
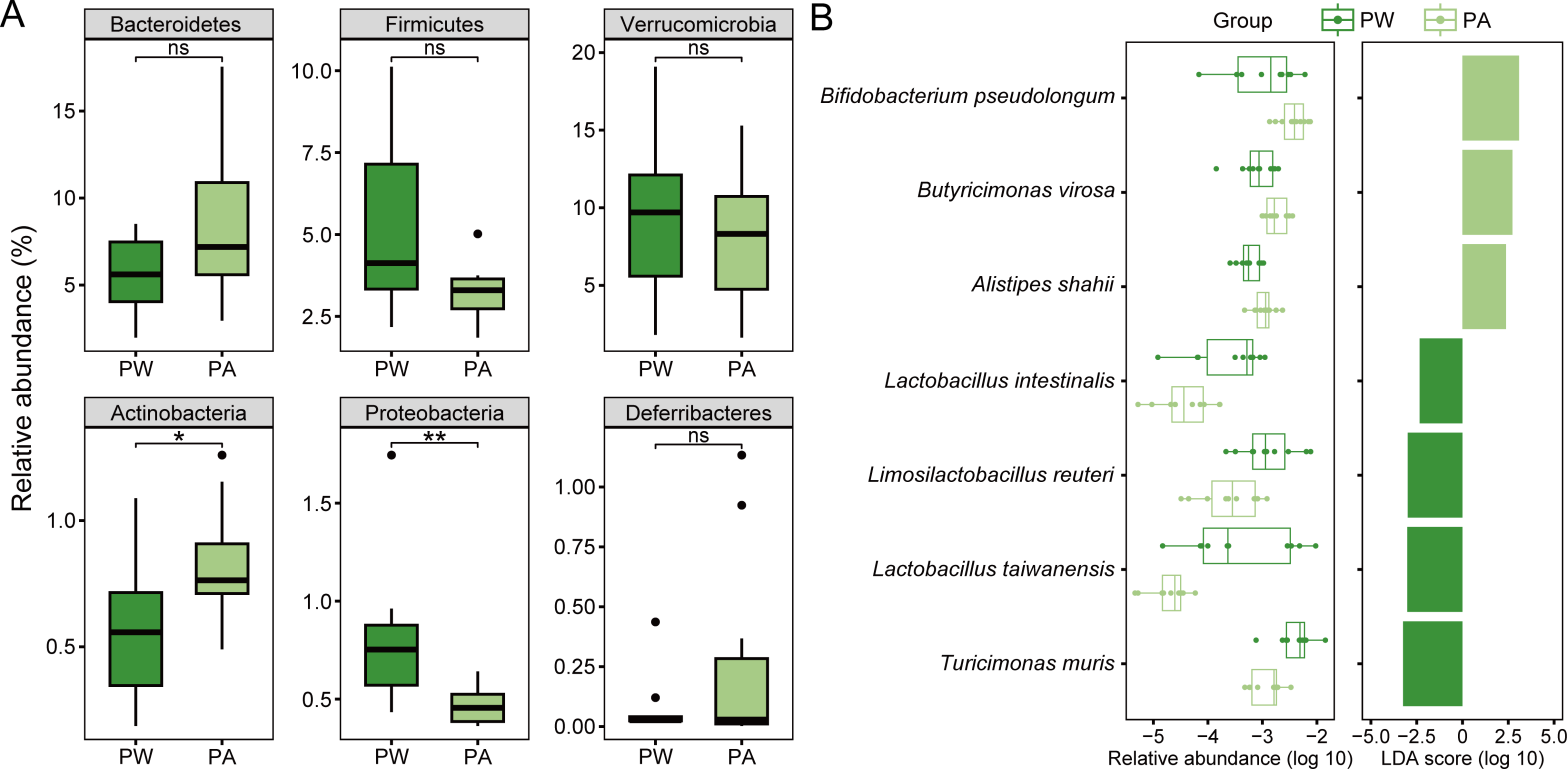
Alterations of gut microbiota in pubertal mice caused by alcohol exposure. (**A**) Relative abundances of Bacteroidetes, Firmicutes, Verrucomicrobia, Actinobacteria, Proteobacteria, and Deferribacteres. (**B**) Seven species with significantly different abundances in the water group and alcohol-exposed group. The left panel shows the relative abundance of the species in each group, and the right panel shows the LDA score. Group: PW, pubertal mice in the water group; PA, pubertal mice in the alcohol-exposed group. Significance levels: *, *P <* 0.05; **, *P <* 0.01; ns, not significant.

Similarly, we conducted a study to investigate the impact of alcohol exposure on gut microbes in pubertal mice. Regardless of alcohol exposure, Bacteroidetes, Verrucomicrobia, and Firmicutes also dominated the gut microbes of pubertal mice, accounting for an average of 35.99%, 34.13%, and 24.04% of the bacterial sequences, respectively. In contrast to the results observed in adult mice, we found that alcohol exposure during adolescence led to a significant decrease in the relative abundance of Proteobacteria while simultaneously increasing the relative abundance of Actinobacteria (Fig. 5A). Furthermore, we did not observe any significant differences in the diversity and composition between the alcohol-exposed group and the water group (Fig. S4A and B).

We observed responses consistent with adult mice at both the genus and species levels. Alcohol exposure significantly decreased the relative abundance of *Lactobacillus* and *Limosilactobacillus*, as well as their members, *Lactobacillus intestinalis* and *Limosilactobacillus reuteri*, while increasing the relative abundance of *Bifidobacterium*, *Butyricimonas* and *Bifidobacterium pseudolongum*, *Butyricimonas virosa*, and *Alistipes shahii* (Fig. 5B; Fig. S4C). Although alcohol changed the intestinal microbiota of pubertal mice less than that of adult mice, some uniquely responsive members, such as *Turicimonas*, *Turicimonas muris*, and *Lactobacillus taiwanensis*, were significantly reduced only in pubertal mice under the influence of alcohol (Fig. 5B; Fig. S4C).

In the observations of four different exposure periods, we found that consistent with adult mice, the alpha diversity of intestinal microorganisms in pubertal mice also showed a trend of first decreasing and then increasing during the observation period (Fig. S5A), and 24 hours of alcohol exposure significantly changed alpha and beta diversity (Fig. S5A and C). However, there were no significant differences in community composition except at the time point of T3 (Fig. S5B). In addition, during the whole observation period, the microbial communities of both groups deviated from the baseline first, but the microbial composition of mice without alcohol exposure tended to be stable after 10 days, while alcohol exposure made the intestinal microbial composition tend toward the baseline (Fig. S5D). These findings suggested that alcohol exposure may affect gut microbial maturation in pubertal mice.

**Fig 6:**
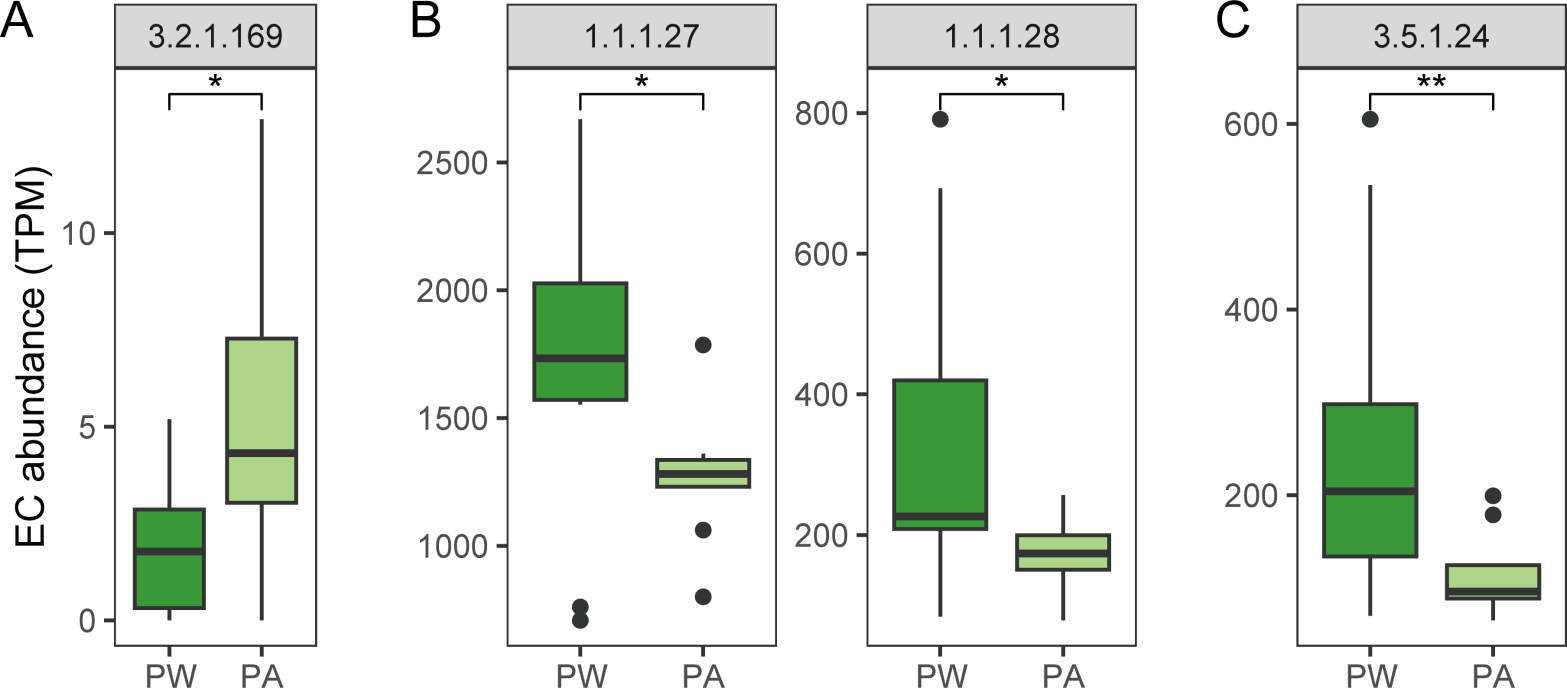
Comparison of the abundance of enzymes involved in (**A**) mucin degradation, (**B**) tryptophan to indoleacetic acid, and (**C**) bile acid conversion in the two groups. TPM (transcript per million) is defined as abundance. Group: PW, pubertal mice in the water group; PA, pubertal mice in the alcohol-exposed group. Significance levels: *, *P <* 0.05; **, *P <* 0.01; ns, not significant. EC: Enzyme Nomenclature (EC number system).

In terms of enzymes, the enzymes involved in mucin degradation were higher in the alcohol-exposed group, while the enzymes that convert bile acids and the synthetase responsible for indole acetic acid production were significantly reduced (Fig. 6A through C).

### Uncovering unique enrichment of novel microbial taxa in response to alcohol consumption

Metagenomic analysis of microbial communities has become an essential tool for understanding the composition and functional potential of complex environments. However, a substantial challenge in such analyses is the presence of a considerable proportion of reads that cannot be assigned to known species. To overcome this limitation and gain a more comprehensive understanding of the microbial diversity, we applied binning techniques to group the reads into MAGs for species-level analysis.

**Fig 7:**
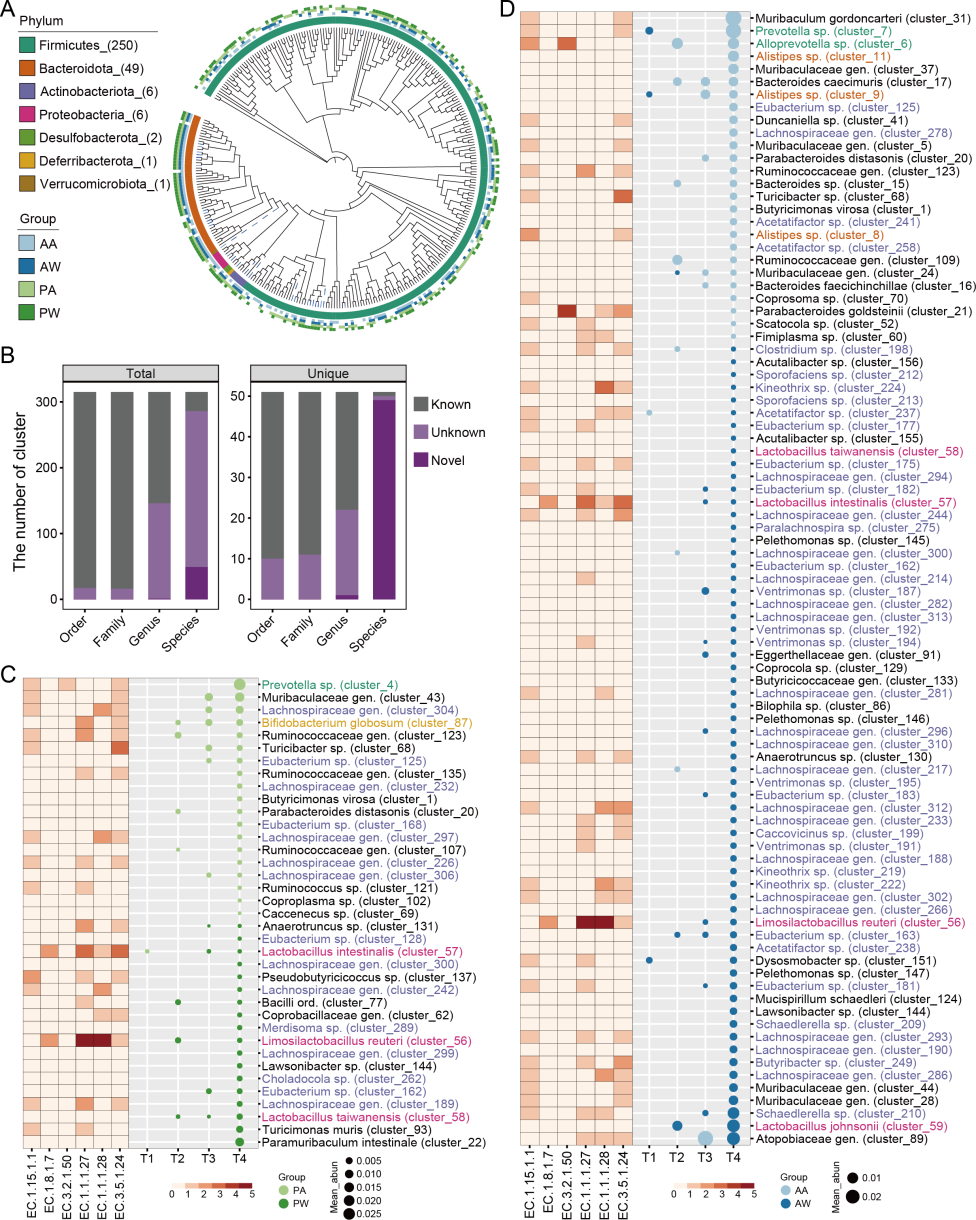
High-quality genome reconstruction from metagenomic data to detect changes in microbial taxa induced by alcohol exposure. (**A**) Phylogenetic tree of the 315 SGBs. Clades of known species are marked as blue dashed lines. The first layer marks the door information, and the second to fifth layers represent the group of samples that generate this SGB. (**B**) Novelty of all 315 SGBs and 51 unique SGBs. Novel means that no genome in GTDB matches it, that is, a completely new SGB. Unknown means that there are genomes that can match but no species name. (**C, D**) SGBs with significantly different abundances in the water group and alcohol-exposed group in the pubertal (**C**) and adult (**D**) mice. The heat map represents the distribution of enzymes in each SGB. The SGBs mentioned in the results are highlighted with different color labels. Group: PW, pubertal mice in the water group; PA, pubertal mice in the alcohol-exposed group; AW, adult mice in the water group; AA, adult mice in the alcohol-exposed group.

Firstly, we utilized three popular binning methods, namely, MetaBAT2, MaxBin2, and CONCOCT, to bin each sample. This approach allowed us to generate a total of 47,367 MAGs, which were then subjected to quality control and clustering procedures. The outcome was a collection of 315 SGBs that were selected for further analysis (Fig. 7A). The 315 SGBs were distributed between seven phyla. Firmicutes (250 SGBs) dominated, followed by Bacteroidota (49 SGBs), Actinobacteriota (6 SGBs), Proteobacteria (6 SGBs), Desulfobacterota (2 SGBs), Deferribacterota (1 SGB), and Verrucomicrobiota (1 SGB). Furthermore, it is worth noting that the majority of the SGBs, a striking 91.11%, remain unknown and have not been previously characterized. The information brought by these unknown species is likely to have been overlooked in the above analyses.

To assess the extent of unique microbial taxa brought about by alcohol intake, we performed a comparative analysis between 315 SGBs from our data set and MGBC, which comprises a comprehensive collection of isolate genomes and MAGs from mouse gut microbes (46). Our comparative analysis revealed 51 SGBs that were exclusively identified in our data set (Fig. S6). Among these, nine unique SGBs were contributed by the water group, while the alcohol-exposed group contributed 20 unique SGBs (Fig. S6), and intriguingly, the majority of these represented novel and uncharacterized species (Fig. 7B). In both pubertal and adult mice, the study identified SGBs with significantly different abundances between the water group and the alcohol-exposed group, as depicted in the heat map, which illustrates the enzyme distribution within each SGB, with those mentioned in the results being accentuated by distinct color labels (Fig. 7C and D). This finding suggests that alcohol intake has the potential to significantly alter the gut microbiota’s composition, leading to the emergence of previously unknown microbial taxa. Additionally, we investigated the genetic diversity contributed by our SGBs in terms of the number of unique genes present in our data set. Our SGBs significantly contributed an additional 557,420 genes (Fig. S6), further expanding the genetic composition within the gut microbiota. This suggests that alcohol intake may not only influence the abundance of specific microbial taxa but may also have a profound impact on the functional potential and genetic diversity of the gut microbiota.

## DISCUSSION

Alcohol exposure is a powerful interference factor of a series of pathological changes and gut microbiota (47, 48). In this study, we found that alcohol exposure damaged the intestinal barrier, especially in the initial intestinal segment. In the case of intestinal barrier damage, the invasion of harmful bacteria such as *Alistipes* will lead to the impairment of immune function (49). The compromised barrier facilitates the translocation of pathogenic bacteria and their endotoxins into the systemic circulation, triggering an immune response characterized by the activation of pattern recognition receptors and the subsequent release of pro-inflammatory cytokines (49). Meanwhile, we observed the disturbance of gut microbiota caused by alcohol exposure in adulthood mice. The *Lactobacillus* and recently reclassified *Limosilactobacillus* decreased, while the relative abundance of the genus *Alistipes* and *Parabacteroides* increased, which was the same as the previous research (50). In addition, enzymes involved in the synthesis of anti-inflammatory substances, such as indoleacetic acid, were significantly reduced in the alcohol-exposed group. Our analysis also highlighted an enrichment of the synthesis pathways related to lipopolysaccharide (LPS) and biotin, which possibly was through the intestinal wall once intestinal permeability increases.

After alcohol exposure, the pubertal mice exhibited distinct compensatory responses to ethanol metabolism compared with the adult mice. Specifically, we observed a decrease in the level of ADH and an increase in the level of ALDH in the liver of the pubertal mice. This shift in enzyme expression may play a crucial role in mitigating the toxic effects of acetaldehyde, a harmful byproduct of ethanol metabolism. The reduction in ADH levels could potentially slow down the conversion of ethanol to acetaldehyde, thereby decreasing the accumulation of this toxic intermediate. Concurrently, the elevated ALDH levels would enhance the conversion of acetaldehyde to acetate, a less harmful substance, thus providing a protective mechanism against acetaldehyde toxicity (51). These findings suggest that pubertal mice may have an adaptive metabolic response to alcohol exposure that differs from that of adult mice, potentially influencing the overall impact of alcohol on their physiology and gut microbiota.

A notable observation showed different results to adult mice that alcohol exposure during adolescence led to a significant decrease in the relative abundance of Proteobacteria while simultaneously increasing the relative abundance of Actinobacteria. Although alcohol changed the gut microbiota of the pubertal mice less than that of the adult mice, some uniquely responsive members, such as *Turicimonas muris* and *Lactobacillus taiwanensis*, were significantly reduced only in the pubertal mice under the influence of alcohol. Compared with the pubertal mice, the abundance of mucin degradation-related enzymes significantly increased within the adult mice exposed to alcohol. This phenomenon may compromise the integrity of the intestinal mucus layer, potentially leading to heightened intestinal permeability.

Our study reveals that alcohol intervention significantly upregulates mucin degradation enzymes and oxidative stress enzymes in the gut microbiota. The increased expression of mucin degradation enzymes suggests a potential compromise in gut barrier integrity, as these enzymes break down mucin, a critical component of the protective mucus layer. This degradation may facilitate the translocation of pathogens and toxins, exacerbating alcohol-induced gut barrier damage. Concurrently, the elevated expression of oxidative stress enzymes indicates a heightened oxidative stress response, which can disrupt the balance of the gut microbiota, promoting dysbiosis and systemic inflammation. These findings highlight the crucial roles of mucin degradation and oxidative stress enzymes in mediating the adverse effects of alcohol on gut health. Understanding these molecular mechanisms is essential for developing therapeutic strategies to mitigate alcohol-induced gut damage and maintain gut microbiota balance. Further research is needed to explore these interactions and their broader implications for human health.

To further study the unknown reads, we obtained 47,367 MAGs by assembly method. Then, the quality control and clustering procedures were carried out, and 315 SGBs were collected. We performed a species-resolved metagenomic analysis, which yielded similar but additional conclusions as above. In the adult mice, alcohol exposure resulted in the enrichment of 26 SGBs, primarily comprising *Prevotella* and *Alistipes*, while 63 SGBs were reduced, with *Lactobacillus* being the most impacted (Fig. 5C). Notably, several unknown species of *Lachnospiraceae* were identified, indicating their potential significance in alcohol-induced changes. Functionally, SGBs that were reduced in the alcohol-exposed group showed higher numbers of enzymes involved in indole acetic acid production and bile acid conversion, especially in *Limosilactobacillus reuteri* (cluster_56) and *Lactobacillus intestinalis* (cluster_57). Additionally, mucin-degrading enzymes were specifically annotated in alcohol-enriched *Alloprevotella* sp. (cluster_6) and *Parabacteroides goldsteinii* (cluster_21) (Fig. 5C). For the pubertal mice, 19 SGBs were enriched in the alcohol-exposed group, mainly comprising *Prevotella* and *Bifidobacterium*, while 18 SGBs were decreased, with *Lactobacillus* being significantly affected (Fig. 5D). Similar to adults, a considerable number of unknown *Lachnospiraceae* species were found to potentially play an important role. In addition, specific microbial taxa, including *Ruminococcaceae* gen. (cluster_123), *Turicibacter* sp. (cluster_68), *Eubacterium* sp. (cluster_125), *Butyricimonas virosa* (cluster_1), and *Parabacteroides distasonis* (cluster_20), were enriched in alcohol-exposed groups of both pubertal and adult mice. Conversely, *Lactobacillus taiwanensis* (cluster_58), *Eubacterium* sp. (cluster_162), *Lawsonibacter* sp. (cluster_144), *Limosilactobacillus reuteri* (cluster_56), *Lachnospiraceae* gen. (cluster_300), and *Lactobacillus intestinalis* (cluster_57) were consistently reduced in both life stages. Functionally, *Limosilactobacillus reuteri* (cluster_56) and *Lactobacillus intestinalis* (cluster_57) exhibited higher levels of enzymes involved in indole acetic acid production and bile acid conversion in response to alcohol exposure, while mucin-degrading enzymes were specifically annotated in alcohol-enriched *Prevotella* sp. (cluster_4) (Fig. 5D). Interestingly, *Lactobacillus* species have been shown to have potential to protect against alcohol-related diseases, and indole and its derivatives may have played a crucial role in it (47, 52–55). These results once again underscore the key protective role of *Lactobacilli* in the gut from alcohol. Our findings demonstrate distinct gut microbiota responses to alcohol exposure in pubertal and adult mice, with both unique and shared microbial changes observed. Notably, *Lactobacillus* consistently emerged as key protectors against the impact of alcohol on the gut. Although pubertal mice have unique MAGs and stronger resilience, they may still have irreversible damage in brain development, which needs further attention.

In future research, we need to pay attention to more organ damage, such as liver, brain, and so on, especially in pubertal mice. Alcohol exposure affects the changes of the gut microbiota, and harmful bacteria may affect the occurrence and development of disease through metabolites, which needs further study. In addition, we should do more research on the impact of alcohol intervention on the gut microbiota in future studies. In summary, our findings illuminate the multifaceted impact of alcohol exposure on the gut microbiota, emphasizing the varying effects across developmental stages, the alteration of microbial composition and metabolic pathways, and the potential consequences on gut barrier integrity, inflammation, and novel microbial taxa discovery.

## Supplementary Material

Reviewer comments

## Data Availability

The metagenomic shotgun sequencing data for all samples have been deposited into CNSA of CNGBdb with accession number CNP0004535.

## SUPPLEMENTAL MATERIAL

Supplemental Figures msystems.01366-24-s0001.docx Figures S1 to S6.

Table S1 msystems.01366-24-s0002.xlsx Primer sequences used in this study.

Table S2 msystems.01366-24-s0003.xlsx Histological scoring system for evaluation of intestinal mucosal damage.

Table S3 msystems.01366-24-s0004.xlsx Statistical analysis for the data presented in the paper.

OPEN PEER REVIEW reviewer-comments.pdf An accounting of the reviewer comments and feedback.

ASM does not own the copyrights to Supplemental Material that may be linked to, or accessed through, an article. The authors have granted ASM a non-exclusive, world-wide license to publish the Supplemental Material files. Please contact the corresponding author directly for reuse.
