# Supplementary material for: Gut microbiota dysbiosis induced by alcohol exposure in pubertal and adult mice
Source: mSystems. 2024 Nov 27;9(12):e01366-24. doi: 10.1128/msystems.01366-24 (PMC11651099; doi:10.1128/msystems.01366-24)
Supplement: Supplemental Figures — Figures S1 to S6. [file msystems.01366-24-s0001.docx]

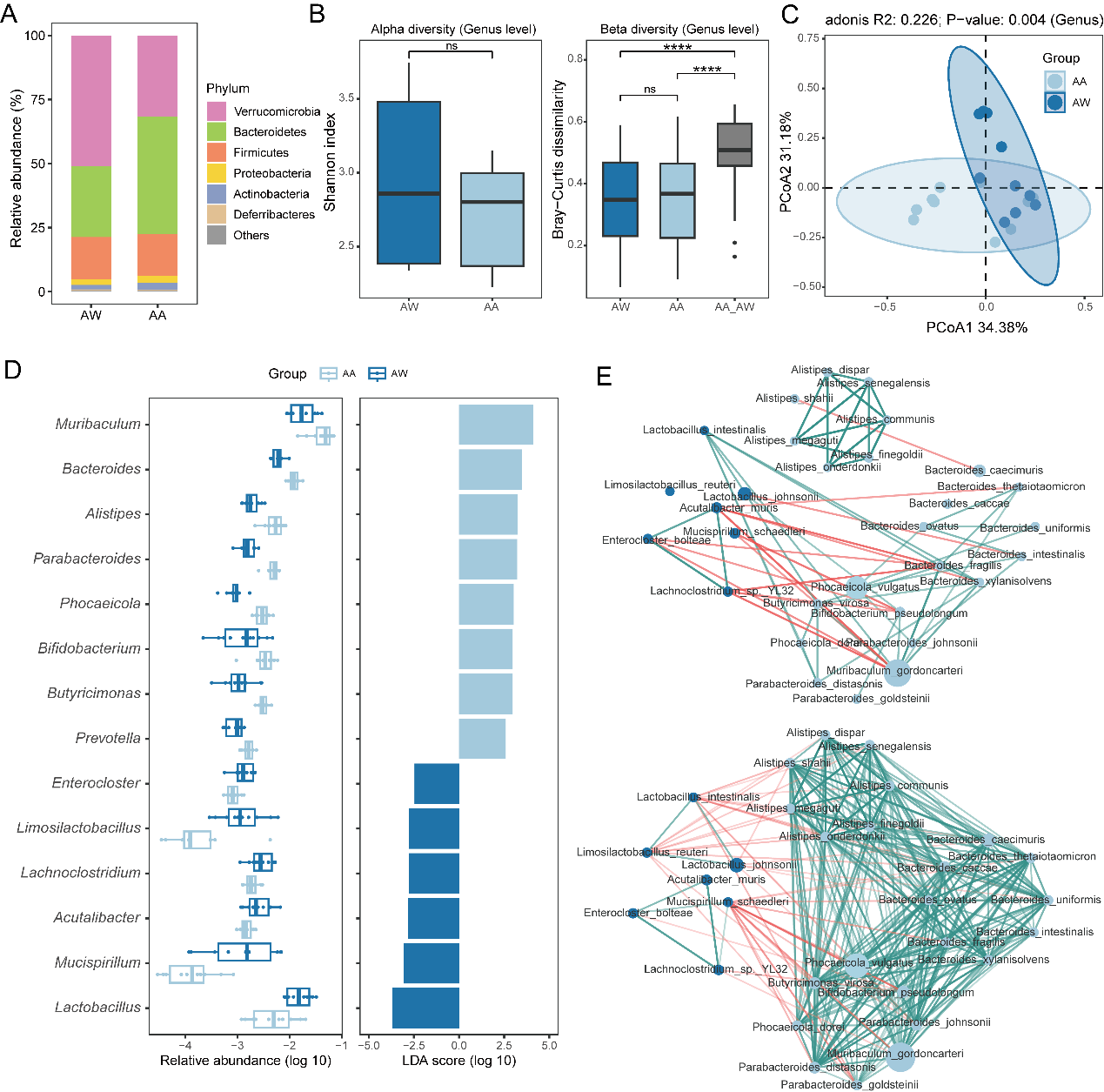


**SFigure 1:** Gut microbial composition of adult mice. (A) Relative abundance of each bacterial phylum. (B) Shannon index and Bray-Curtis dissimilarity of gut microbiota at the genus level from each group of mice. (C) Principal coordinate analysis (PCoA) of gut microbiota from each group of mice. (D) 14 Genus with significantly different abundances in the saline group and alcohol-exposed group. (E) Co-abundance network of 29 species with significantly different abundances. The color of the dot indicates the enriched group, and the size indicates the average abundance in that group. Dark green lines indicate significant positive correlations, while red lines indicate significant negative correlations.


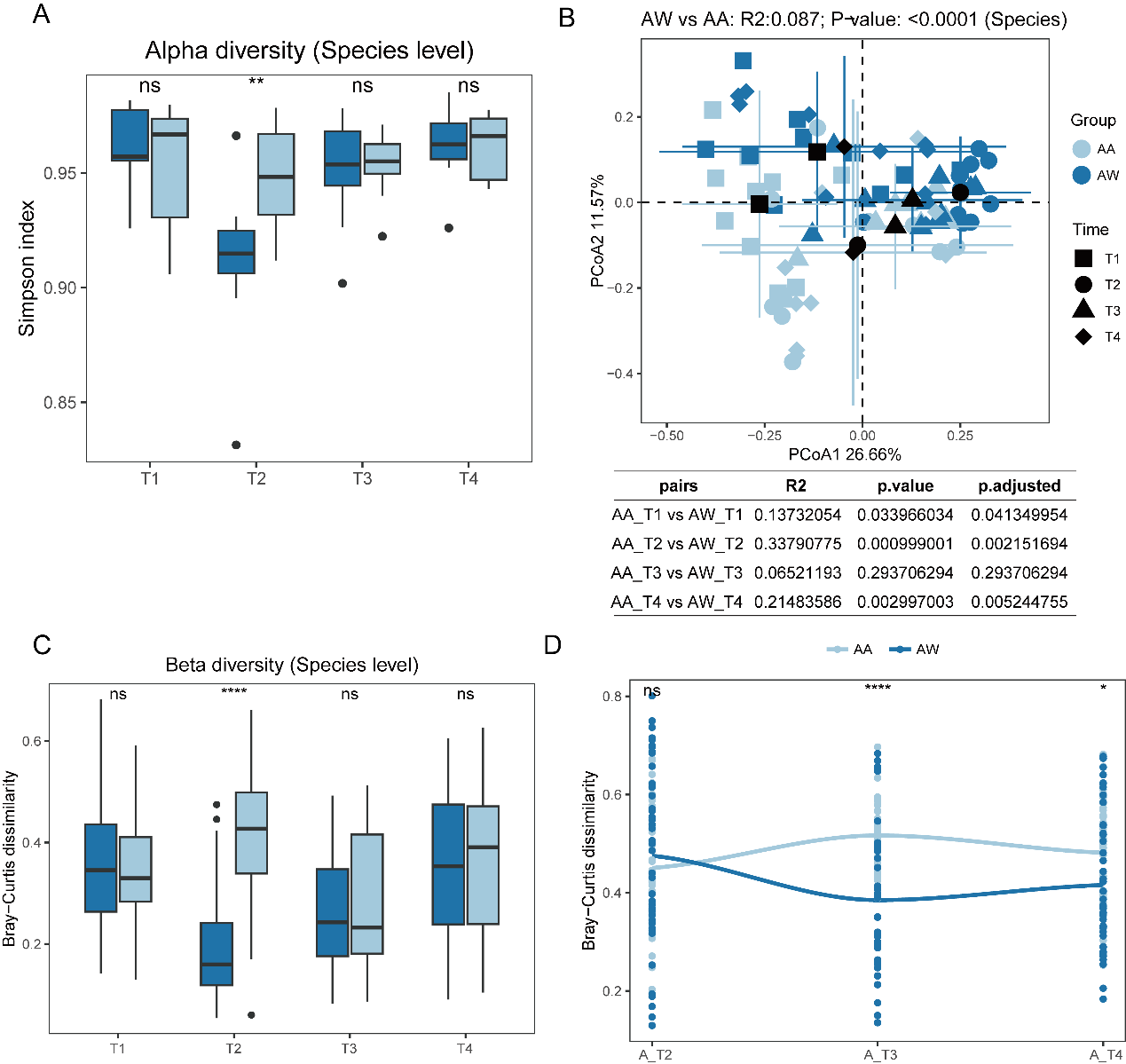


**SFigure 2:** Alpha diversity (A), Beta diversity (B), PCoA (C), and Bray-Curtis dissimilarity (D) of gut microbiota at the species level from each group of adult mice during the exposure periods.


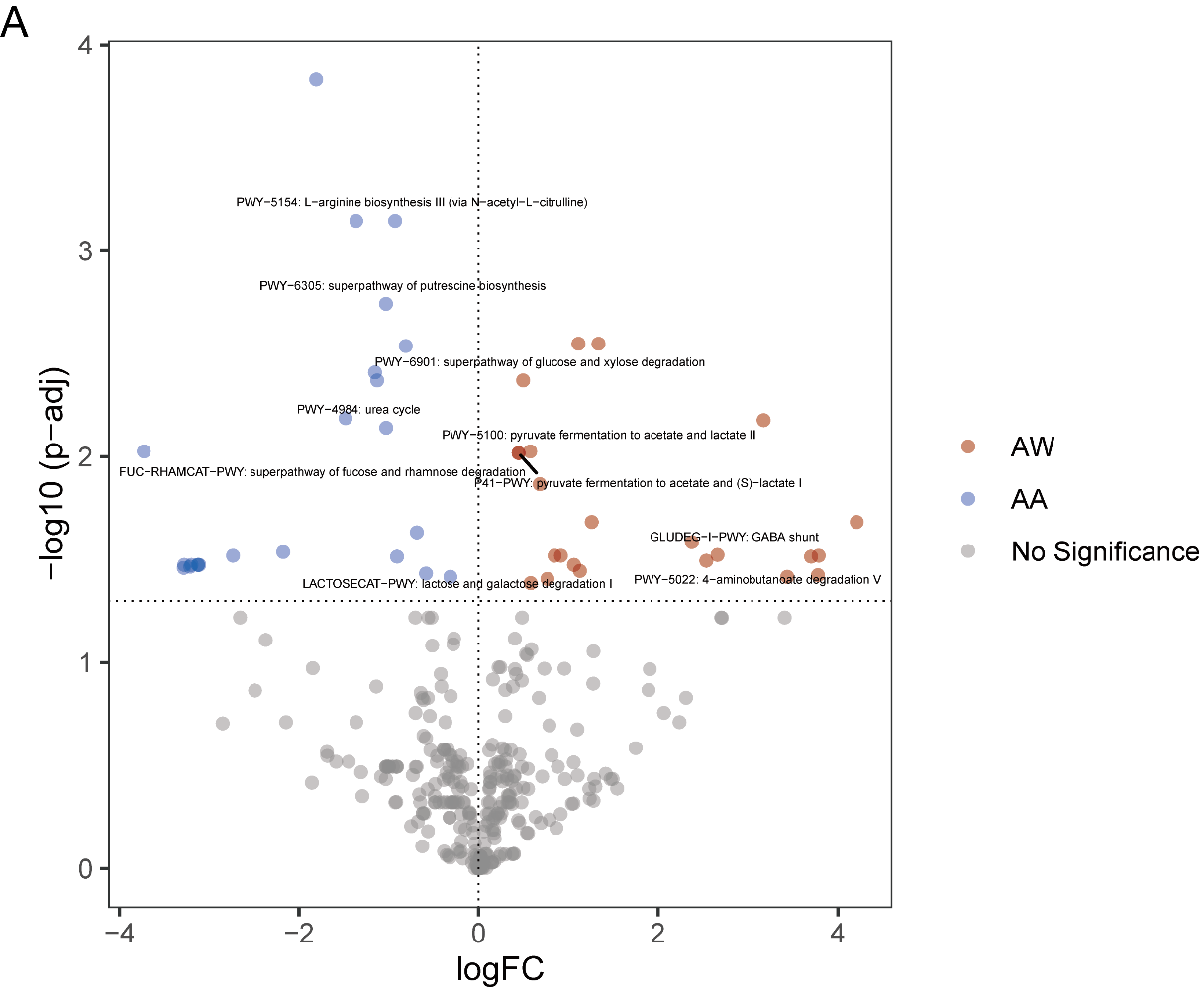


**SFigure 3:** MetaCyc pathways differentially enriched in saline group and alcohol-exposed group.


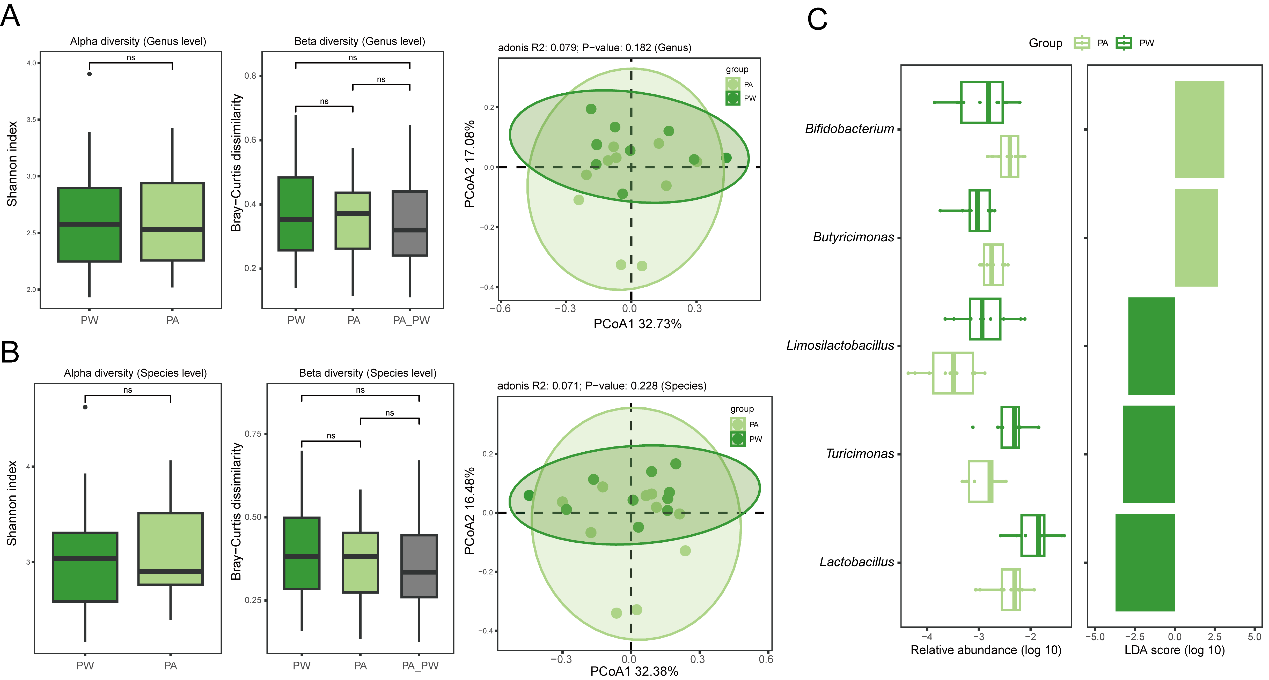


**SFigure 4:** Gut microbial composition of adolescent mice. (A and B) Shannon index, Bray-Curtis dissimilarity, and Principal coordinate analysis (PCoA) of gut microbiota at the genus (A) and species level (B) from each group of mice. (C) 5 Genus with significantly different abundances in the saline group and alcohol-exposed group.


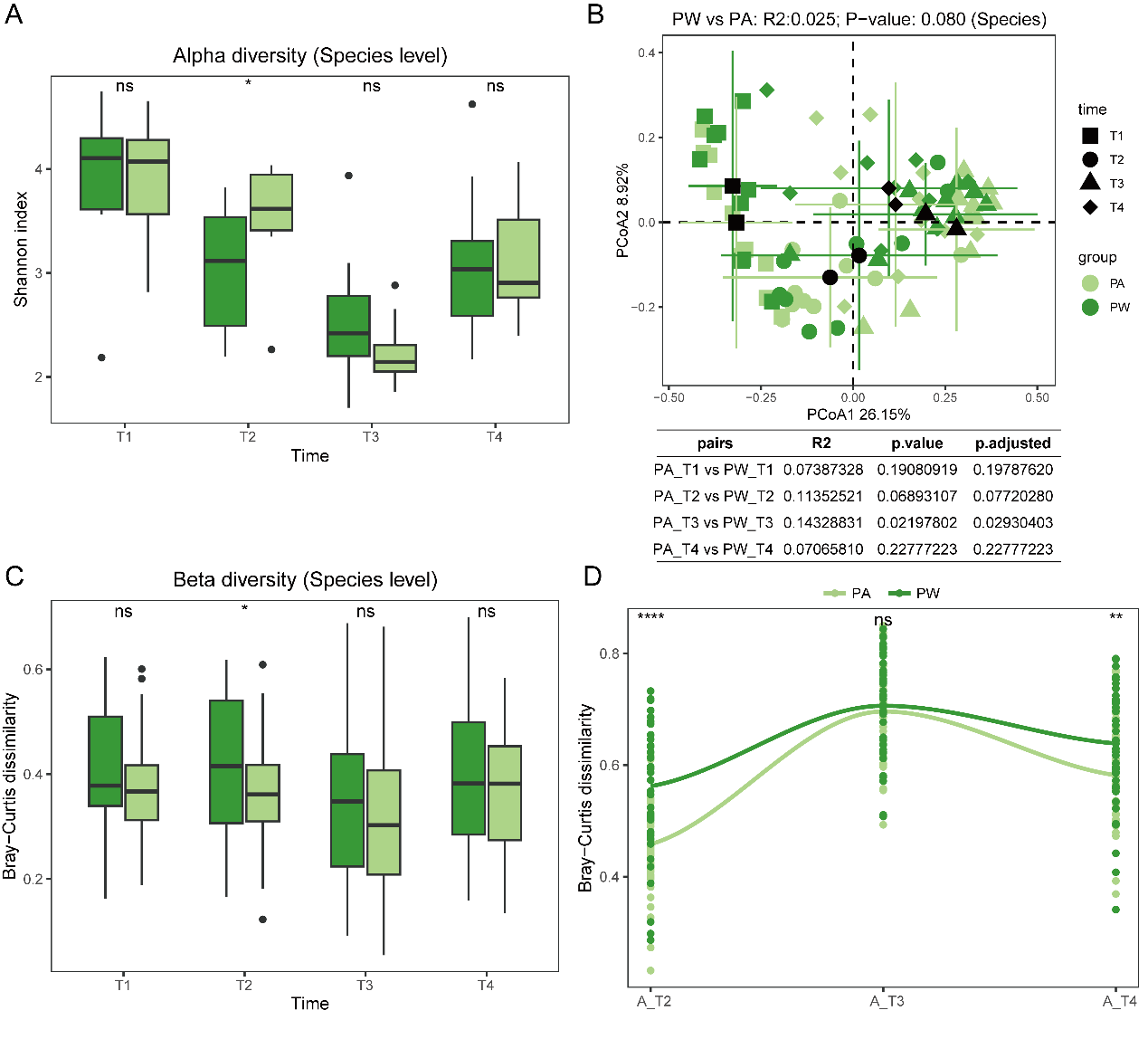


**SFigure 5:** Alpha diversity (A), Beta diversity (B), PCoA (C), and Bray-Curtis dissimilarity (D) of gut microbiota at the species level from each group of adolescent mice during the exposure periods.


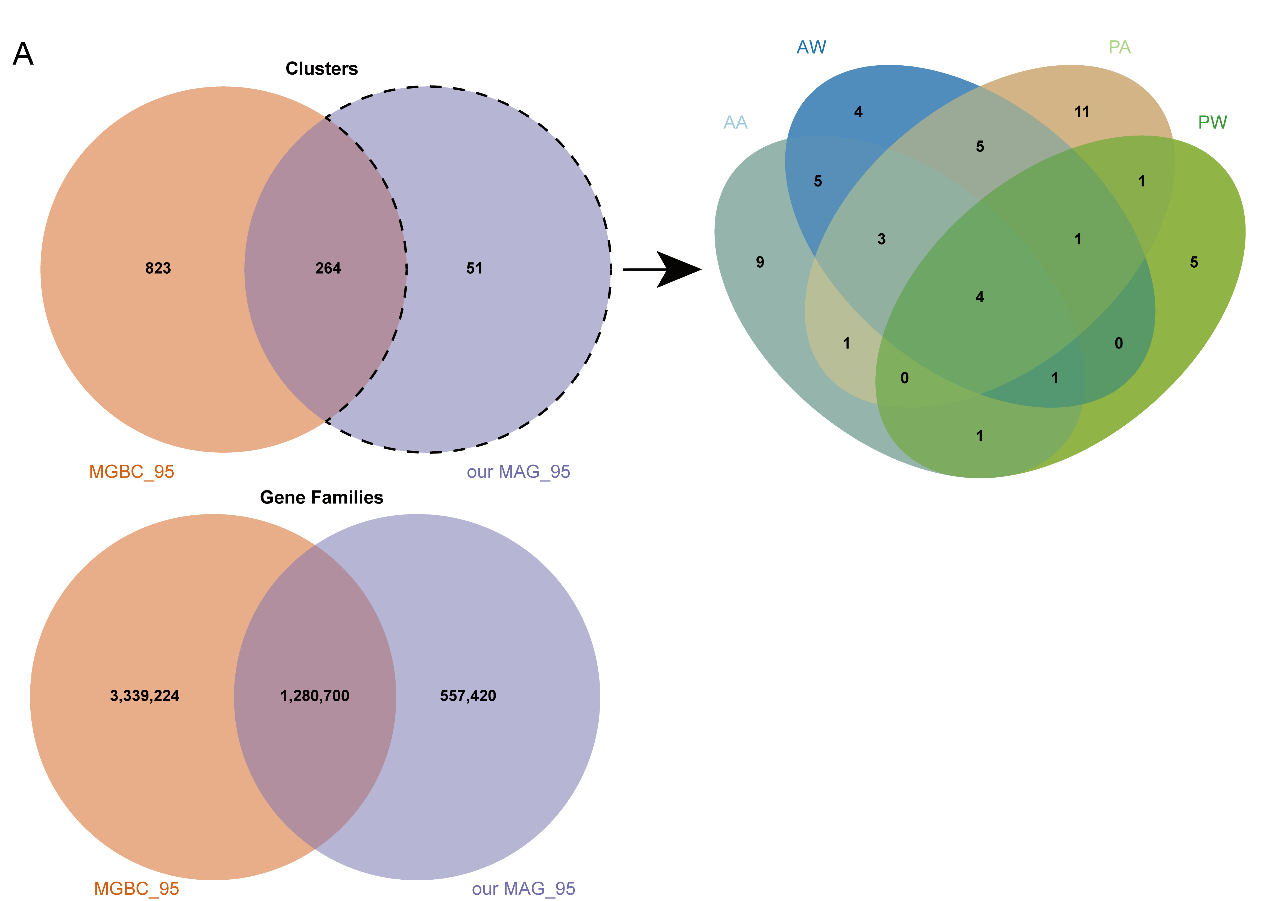


**SFigure 6:** Comparison with MGBC at cluster and gene families, respectively.
